# The Alternative to State Aid. Local Voluntary Effort

**Published:** 1919-12-20

**Authors:** Henry C. Burdett

**Affiliations:** 43 Porchester Terrace, W. 2.


### HOSPITAL FINANCE.

### The Alternative to State Aid, Local Voluntary Effort.

*To the Editor of* The Times.

Sir,—When the King's Hospital Fund was founded in 1897 to commemorate the Diamond Jubilee of Queen Victoria, King Edward VII., then Prince of Wales, wrote a letter to the public, dated February 5 of that year, in which he inaugurated the Fund, as recorded in *The Times* of the 13th inst. In the course of it he wrote:—

> I have been struck by the statement, the truth of which is placed beyond doubt, that the contributors to the funds of our hospitals number less than one in a hundred of the population.

As the result of later investigations, and a close examination of the condition of matters in this respect to-day, I have good reason to state that in practically every city or town, still, some nine-tenths of the whole population give nothing whatever to a hospital, or to the aid of the injured and the sick. This is true not of one city but of all cities, not of one town but of all towns and counties throughout Great Britain.

How can this state of affairs be remedied with the minimum of difficulty and the maximum of speed? Already the country is awakening to the danger with which the people are threatened in regard to our hospital system; and the centres of population, as represented by great cities like Liverpool, and the country generally by smaller but important cities like Derby, Oxford, Stamford, and others, have taken steps to raise large sums to tide over the difficult times which may lie ahead. I give another three notable instances sent to me. The Nottingham General Hospital asked for £100,000 as a war memorial. Already in a few weeks £92,000 has been subscribed. They require £20,000 increased income. The workpeople, led by the miners, have doubled their weekly contributions and have agreed to find an extra £10,000 yearly for income. The remainder of the population will find the other half needed.

Next take a county hospital, Bedford. The chairman, Mr. Whitchurch, writes:—

> All parts of the community have helped us generously. We supply a clear statement of what we want and how the money is to be spent. We trust them. They trust us.

They asked for £8,000 for maintenance and £8,000 for a convalescent home of twenty beds. The answer to December 11 was £8,000 for maintenance and already £7,200 for a convalescent home.

**Hospital Finance**—(*continued from p.* 268.)

Further, the nurses' home required rebuilding, and £3,000 is already in hand.

Next I take a North Country hospital, Warrington Infirmary. They wanted £4,000 for extensions to the nurses' home, to increase the staff and reduce the nurses' hours on duty, and generally to increase subscriptions: £10,000 has been raised at once.

It remains for every city, town, and county to arouse and to put its house in order by following these excellent examples. Every voluntary hospital everywhere has in its midst armies of active, young, interested, and loyal supporters, in the shape of the men and women who have been demobilised, and who know by experience the inestimable value and necessity of maintaining the voluntary system. With their co-operation every community can be organised with the minimum of trouble and the maximum of efficiency. There is plenty of money, and there are plenty of people willing to give it to the hospitals, providing that the needs and dangers and the amounts required are put plainly before them and an active canvass organised, so that the whole community in every centre may be fully apprised of the facts and aroused to do its duty without delay. Realising this, it has been my privilege to be in communication with the chairmen of all the voluntary hospitals with 100 beds and upwards throughout Great Britain. It is only four days since my letter reached the chairmen, and I already have replies from the most representative and important hospitals in England and Scotland gratefully welcoming the step taken with a view to bringing this new organisation everywhere throughout the country into active being. Those chairmen who have not replied will, I hope, make a point of seeing that they receive my letter in due course, and kindly co-operate.

The only impediment which is causing many even of the habitual givers to our hospitals to hold their hand at the moment is the silence of the Government, and especially the Prime Minister, who, it is feared, are represented by certain clamorous agitators to favour the establishment of rate-supported hospitals, and the casting of the voluntary hospitals on the rates, although all British statesmen recognise that the State has so much on its hands that any question of the nationalisation of hospitals, apart altogether from the merits or demerits of any such fate, must be postponed into the dim future. Manifest as this is, the silence of the Government creates suspicion, and is causing the withholding of considerable sums of money which would otherwise be handed over to the treasurers of the voluntary hospitals. Legacies are also being withheld for the same reason. What is wanted now is a reassurance from the Government, and especially from two members of it, the Prime Minister and the Health Minister, that the substitution of rate-supported hospitals for the voluntary system is neither feasible nor contemplated.

Both Houses of Parliament contain a large number of members who are zealous supporters of the voluntary system. It need only occupy five minutes of Parliamentary time to put a definite question to the Government in each House, and to obtain a definite and convincing reply, with a guarantee that the voluntary hospitals will not be made the plaything of politicians. I therefore appeal most earnestly to members of both Houses of Parliament to have this matter cleared up without a moment's avoidable delay. I am in a position to give an assurance, which will be enforced by the gratifying statements I have received from hospital chairmen throughout Great Britain, including some of the eminent men of the day, that, providing the necessary machinery is set to work with the New Year on the right lines in every district where there are hospitals, by Midsummer Day, 1920, with God's blessing, the financial crisis of our hospitals will everywhere be solved, and the time for an awakened people, inhabiting the true Merrie England which we all desire, will have come.

I am, yours faithfully,

HENRY C. BURDETT.

43 Porchester Terrace, W. 2.

